# Complete excision of esophageal bronchogenic cyst by endoscopic submucosal tunnel dissection: a case presentation

**DOI:** 10.1186/s12876-019-1072-3

**Published:** 2019-08-28

**Authors:** Xun Yang, Ye Zong, Hai-Ying Zhao, Yong-Dong Wu, Ming Ji

**Affiliations:** Department of Gastroenterology, Beijing Friendship Hospital, Capital Medical University, No.95 Yongan Road, Xicheng District, Beijing, 100050 China

**Keywords:** Esophageal bronchogenic cyst, Endoscopic treatment, Case report

## Abstract

**Background:**

Intramural esophageal bronchogenic cyst is very rare. Surgical removal of the cysts is advised even the patients are asymptomatic, since the cyst can lead to complications, and there is a risk of malignant transformation. Thoracotomy or thoracoscopy is the most commonly used approach for complete excision of the cysts. To our knowledge, this is the first report to excise intramural esophageal bronchogenic cyst completely by endoscopic submucosal tunnel dissection (ESTD).

**Case presentation:**

A 40-year-old male was referred to our hospital due to the detection of a submucosal tumor at the distal esophagus. The tumor was found during gastroendoscopy in a general health check-up. The patient had no symptoms. A benign esophageal tumor was confirmed by endoscopic ultrasonography (EUS) and computed tomography (CT). On the basis of these results, ESTD was performed. During the procedure, a cystic mass was observed between the mucosa and the muscular layers of the esophagus, and a hybrid knife was used for dissection. Histopathological examination showed the cyst wall was lined by pseudostratified ciliated columnar epithelium, consistent with a bronchogenic cyst. The esophagography using meglumine diatrizoate showed no leakage on the seventh day after ESTD. The patient remained asymptomatic and had a regular diet during the follow-up period.

**Discussion and conclusions:**

We successfully utilized ESTD for complete removal of esophageal bronchogenic cysts originating from the muscularis propria. The approach appeared safe, providing a minimally invasive treatment option for patients.

## Background

Bronchogenic cyst is a congenital lesion derived from the primitive foregut, and is one of the most common primary cysts of the mediastinum. Although periesophageal bronchogenic cysts have been frequently reported, a completely intramural cyst is rare [1]. Bronchogenic cysts are usually asymptomatic, but there is a risk of malignant transformation and an increased risk of developing complications in adulthood [2, 3]. A surgical resection is therefore recommended. Thoracotomy or thoracoscopy are the common approaches for the resection in adults [1]. In this report, we share our experiences in treating intramural esophageal bronchogenic cyst by a novel method, endoscopic submucosal tunnel dissection (ESTD). We also provided a review of the literatures on the treatment of esophageal bronchogenic cysts.

## Case presentation

**Fig. 1:**
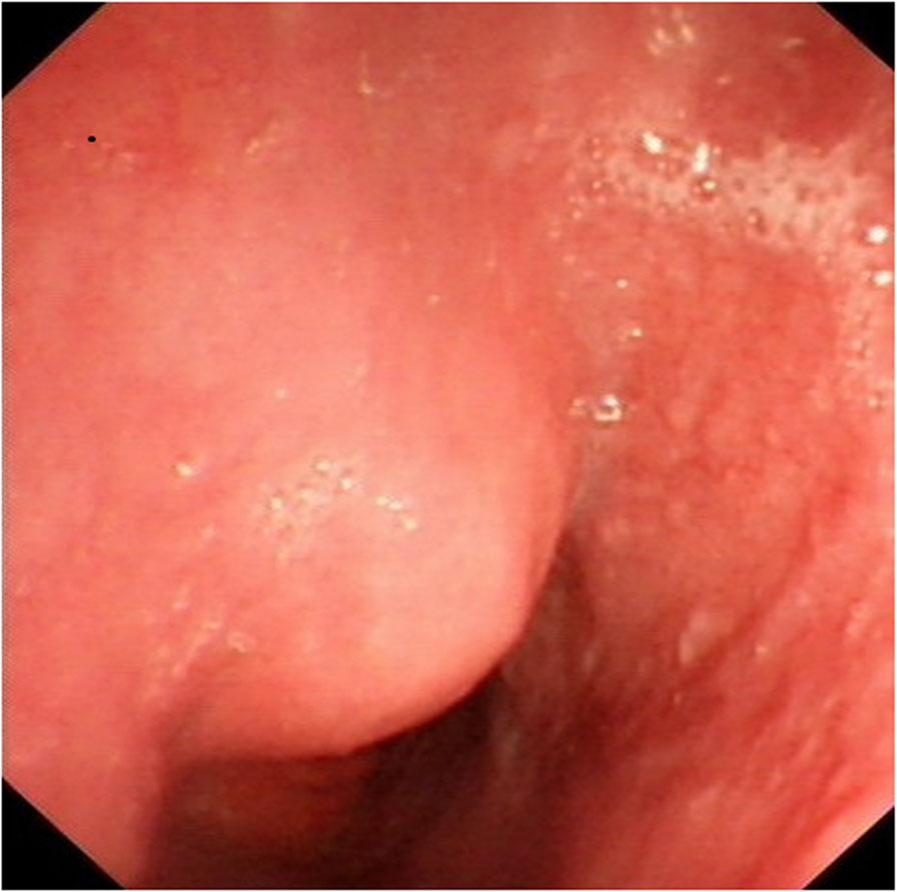
Gastroscopy showed a hemispheric elevation covered by normal mucosa located in the distal portion of the esophagus at 35 cm from the incisors

**Fig. 2:**
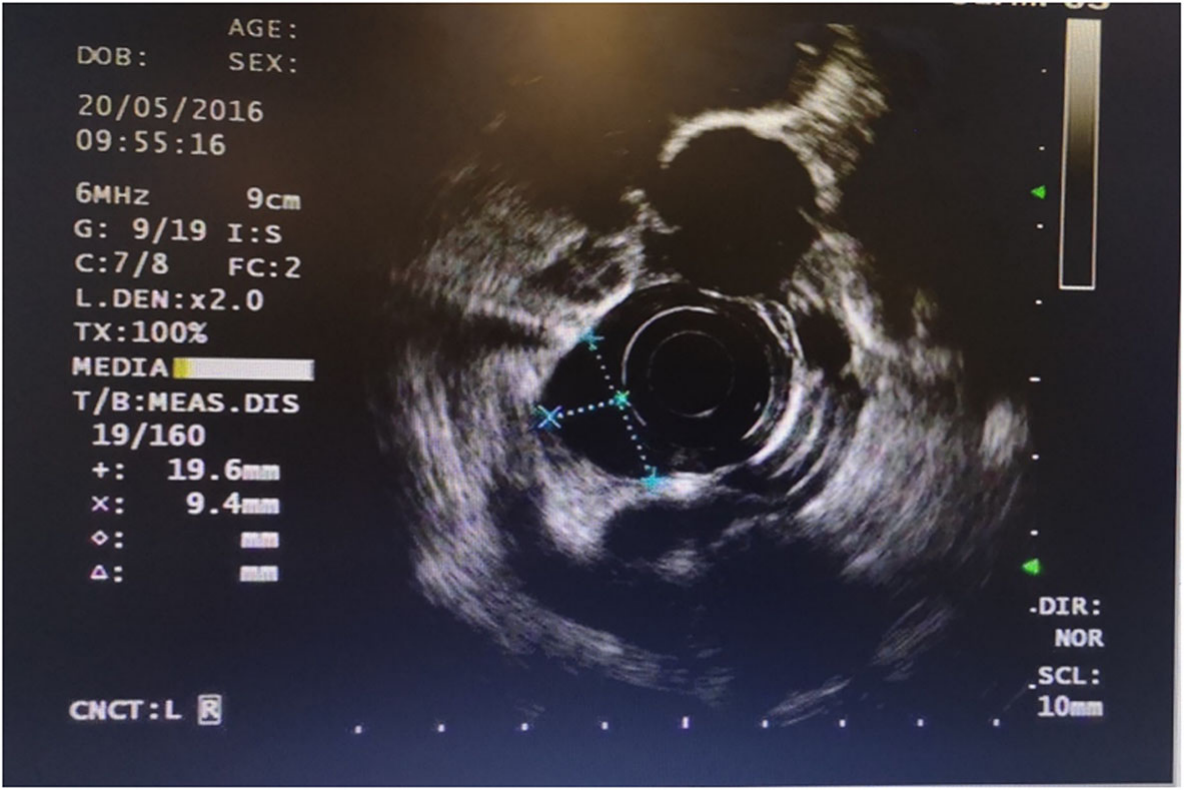
Endoscopic ultrasound showed the anechoic mass arising from muscularis propria, with the size of about 3.0*2.0 cm

**Fig. 3:**
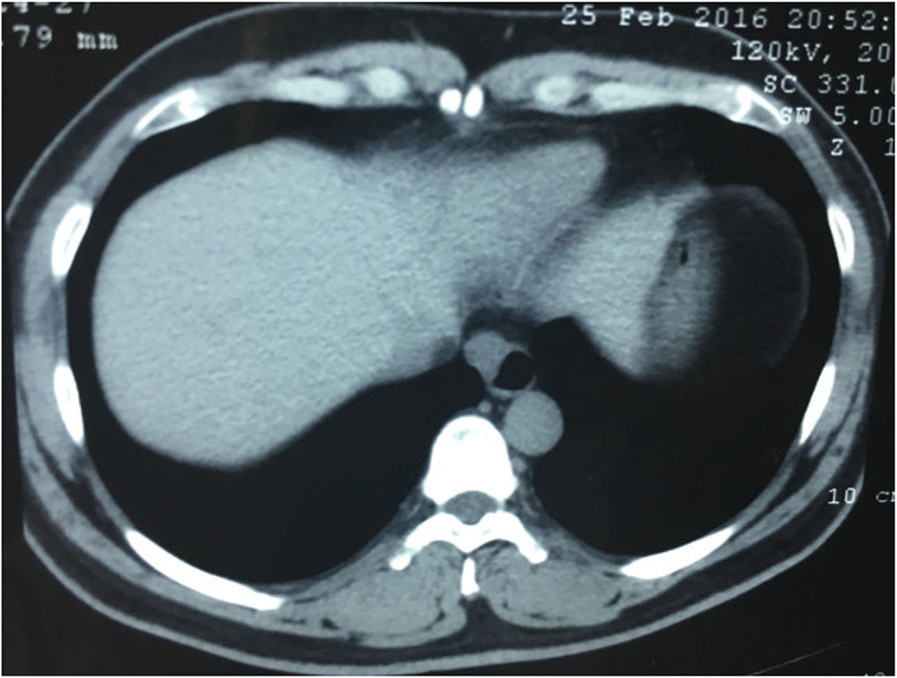
CT demonstrated a soft-tissue attenuation mass from the distal esophageal wall

**Fig. 4:**
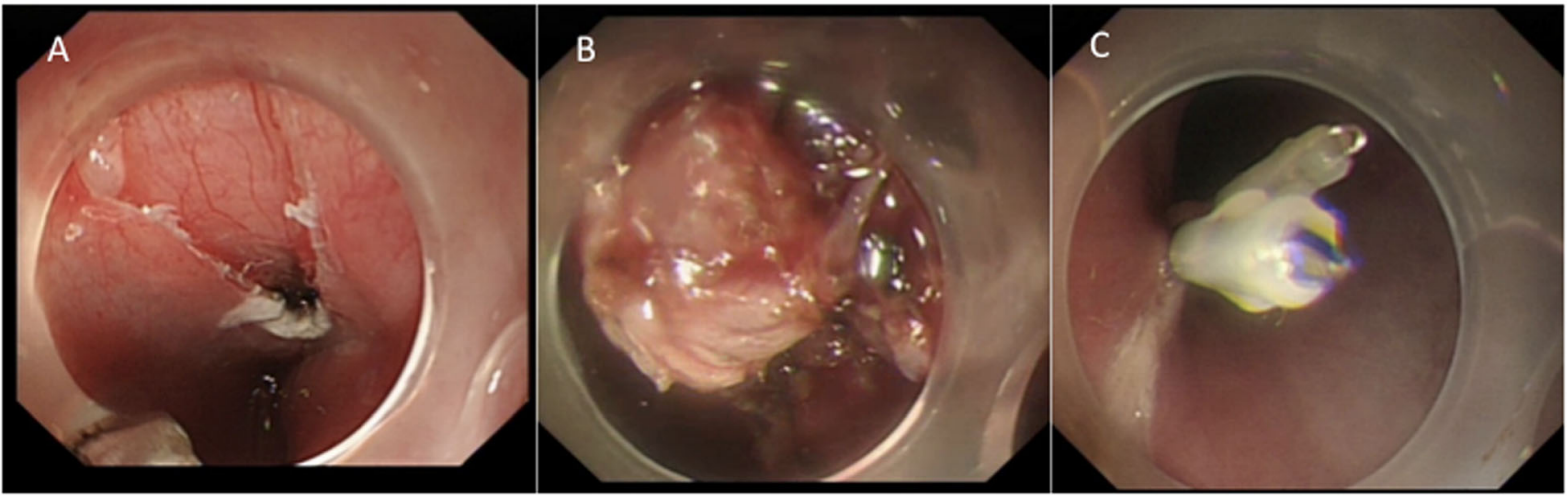
Esophageal bronchogenic cyst excised completely by endoscopic submucosal tunnel dissection. **a** The entrance of the esophageal submucosal tunnel. **b** The esophageal bronchogenic cyst was dissociated. **c** The entrance to the submucosal tunnel was closed by the endoclips

**Fig. 5:**
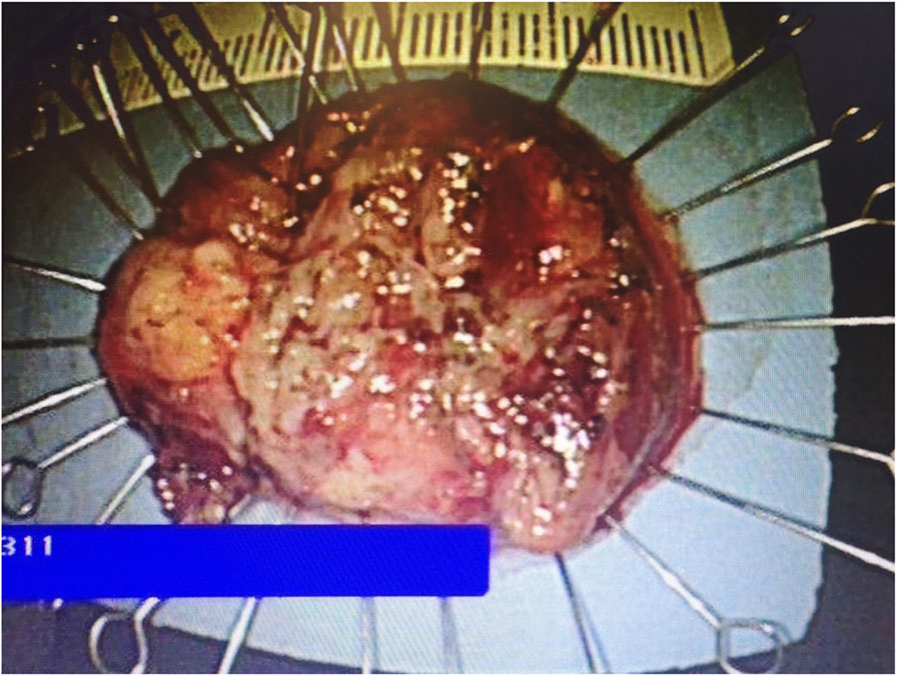
The resected lesion

**Fig. 6:**
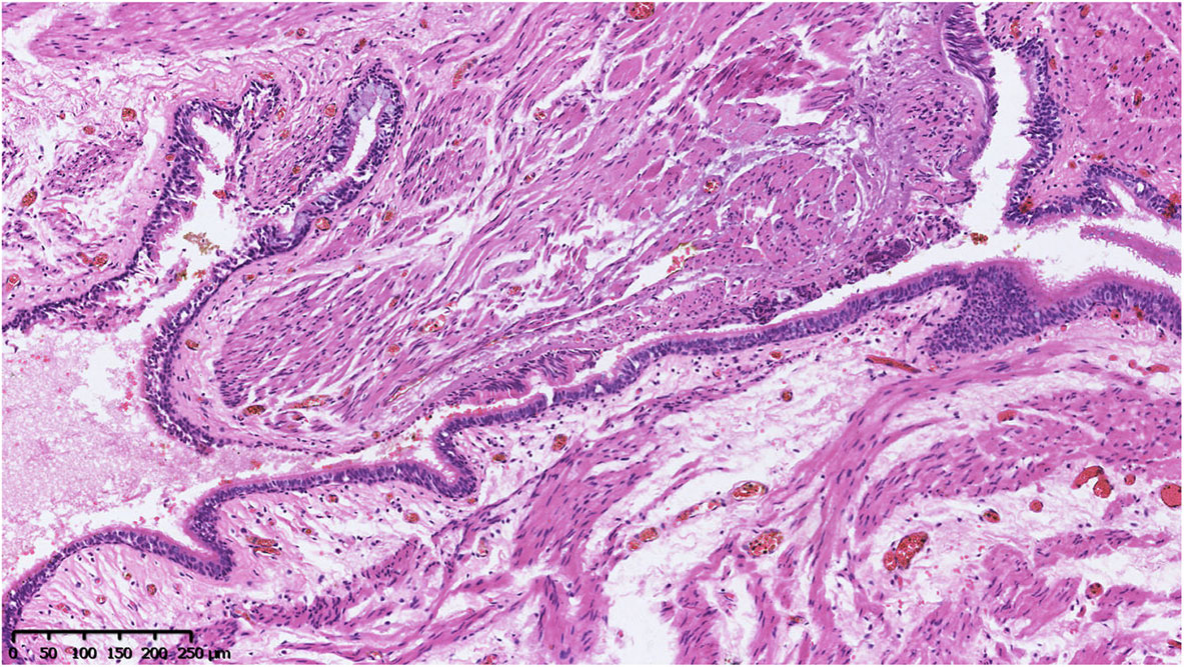
Histopathological examination showed the cyst wall was lined by a pseudostratified ciliated columnar epithelium, consistent with a bronchogenic cyst

**Fig. 7:**
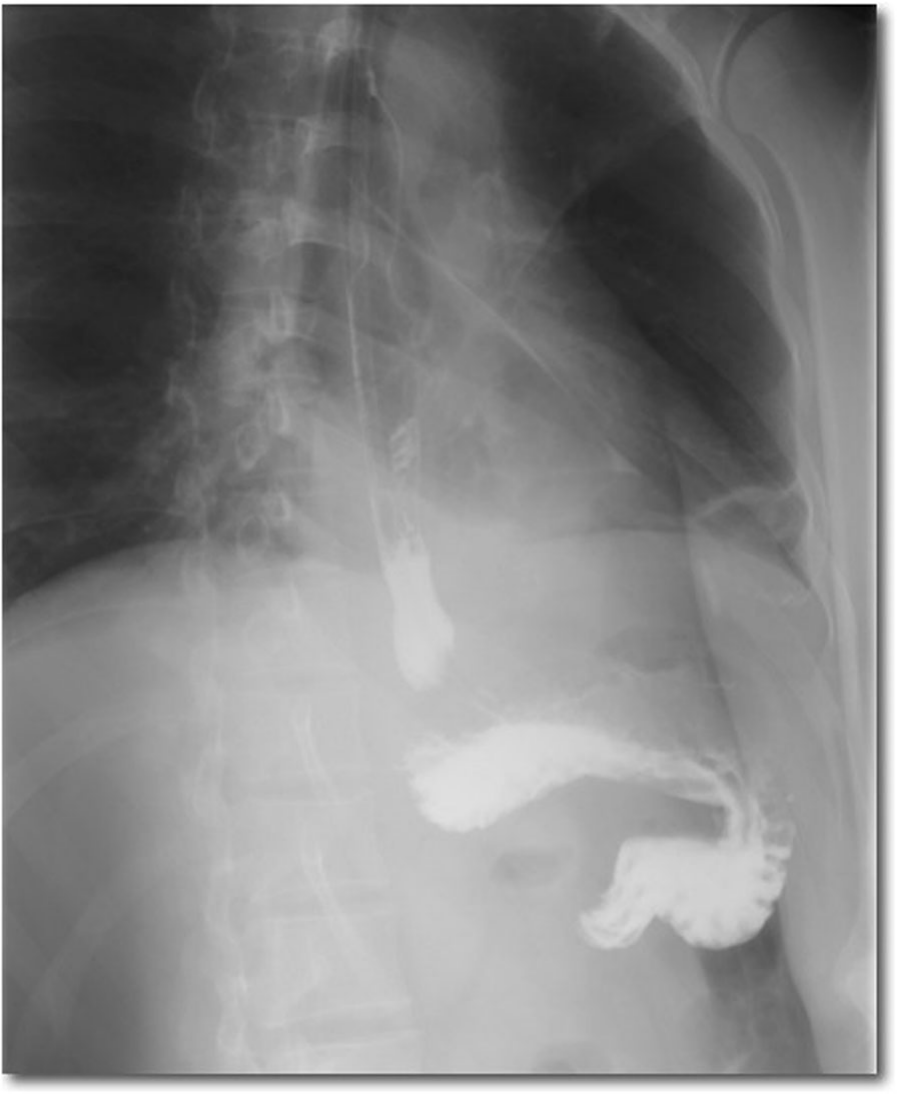
An esophagography with meglumine diatrizoate performed at Day 7 post-ESTD showed no leakage

A 40-year-old male was referred to our hospital due to the detection of a submucosal tumor at the distal esophagus. The tumor was found during gastroendoscopy in a general health check-up. The patient had no dysphagia, chest pain, vomit, fever, cough nor dyspnea. No clinically significant abnormalities were found during physical examination. Laboratory results were all within normal range, which included serum tumor marker (CEA at 2.56 ng/mL; CA19–9 at < 2.00 U/mL), complete blood test, erythrocyte sedimentation rate, hepatic and renal function tests. During the gastroendoscopy, an abnormal swelling covered with normal mucosa was found on the right wall of esophagus at 35 cm from the incisors (Fig. 1). Endoscopic ultrasound (EUS) demonstrated a mass arising from muscularis propria, with a size of about 3.0*2.0 cm (Fig. 2). On computed tomography (CT), the mass was a soft-tissue from the distal esophageal wall. On the basis of these results, a benign esophageal tumor was suspected (Fig. 3), and ESTD was then performed to treat that. However, during the procedure, a cystic mass between the mucosa and muscular layers of the esophagus was observed, and a dissection was performed using the hybrid knife (Fig. 4). The resected lesion was shown in Fig. 5, and Hematoxylin and Eosin (H&E) staining was performed on the specimen. The staining showed that the cyst was lined by a pseudostratified ciliated columnar epithelium, a characteristic consistent with a bronchogenic cyst (Fig. 6). The esophagography with meglumine diatrizoate showed no leakage on the seventh day after ESTD (Fig. 7). The patient remained asymptomatic and had a regular diet during the follow-up period.

## Discussion and conclusions

Bronchogenic cyst is a benign congenital malformation of the mediatisnum, and the cyst can be found in a variety of locations such as paratracheal, carinal, or hilar. Esophageal brochogenic cyst is extremely rare [1, 4]. Most patients with esophageal bronchogenic cysts are asymptomatic, although some may present dysphagia, chest pain and chest discomfort [1, 4]. It is worth noted that in this case benign esophageal tumor was highly suspected on the basis of the laboratory results and clinical examination before surgery, and cystic lesion was not considered at that point. Otherwise, EUS fine-needle-aspiration (FNA) can be an alternative in adding at the diagnosis of the cystic lesion. In addition, the differential diagnosis between esophageal bronchogenic versus duplication cyst can be challenging, and a pathology report is needed for the diagnosis.

Surgical resection of bronchogenic cyst is often recommended, as the cyst can lead to complications including infection, hemorrhage, rupture and malignant degeneration if left untreated [5]. To date, thoracotomy or video-assisted thoracoscopy has been used for the complete surgical excision of the cysts [6–8]. The endoscopic treatment approach is not common. One reason is that the completely esophageal intramural cysts are very rare. In addition, most bronchogenic cysts are located in the superior, middle mediastinum, which are in proximity to the esophagus. Sashiyama et al. first reported the successful use of endoscopic mucosal resection (EMR) to remove an esophageal brochongenic cyst [9]. In that case, the cyst located within submucosal layer, and the submucosal cyst must be elevated by injection of saline solution. If the cyst is located within the muscular layer, EMR cannot be used to resect the lesion. Another case study reported the use of endoscopic submuscosal dissection (ESD) to remove an intra-esophageal bronchogenic cyst confined to the submucosa layer [10]. The lesion in our case located deeper at the muscular layer when compared to the case mentioned. Therefore, the use of ESD in our patient may have a high risk of perforation.

To our knowledge, this is the first report that utilized ESTD to excise the esophageal bronchogenic cyst completely. Tang et al. reported a treatment of esophageal brochogenic cyst with ESTD, the cyst was not completely excised by ESTD [11]. During the procedure, the yellowish milk fluid content was first aspirated, and the cyst wall was excised using endoscopic argon plasma coagulation. Although ESTD is an interventional approach that harbors possible complications, the resection was performed due to the relatively large lesion and young age of the patient. Several reports have indicated the possibility of the malignant transformation of bronchogenic cysts [12, 13].

The use of ESTD allows en bloc resection. A previous report indicated a recurrence of mediastinal cysts 15 years after incomplete resection [14]; therefore a complete removal of the cyst is very important. In our case, ESTD was successful, because the esophageal brochongenic cyst was superficial and small in size. There were not that many esophageal bronchogenic cyst located within the esophageal wall, many of which were located external or in some way connected to the wall. Those cases were difficult to be removed by endoscopic resection. Also, if the bronchogenic cyst is too large and is growing externally to the wall, endoscopic resection cannot be used either.

In our experiences, EUS is sensitive for distinguishing cystic from solid masses. It facilitates the diagnosis of intramural esophageal lesions by providing clear images delineating the size, layer of origin, and location of the cyst relative to esophagus. In summary, we reported the successful use of ESTD to completely remove the esophageal bronchogenic cyst originated from the muscularis propria. The procedure was minimally invasive and appeared safe, although a long-term follow-up for further assessment of the long-term outcome is needed.

## Data Availability

The datasets generated and analyzed during the current study are available from the corresponding author on reasonable request.

## Abbreviations

CT: Computed tomography
EMR: Endoscopic mucosal resection
ESTD: Endoscopic submucosal tunnel dissection
EUS: Endoscopic ultrasonography
